# Targeted Endoscopic Therapies for Gastro-Esophageal Reflux Disease (GERD): A Narrative Review

**DOI:** 10.3390/jpm16040190

**Published:** 2026-04-01

**Authors:** Pier Alberto Testoni, Sabrina Gloria Giulia Testoni

**Affiliations:** 1 Faculty of Medicine and Surgery, Vita-Salute San Raffaele University, 20132 Milan, Italy; 2 Department of Health Sciences, University of Milan, 20122 Milan, Italy; sabrina.testoni@unimi.it

**Keywords:** endoluminal anti-reflux therapy, gastro-esophageal reflux disease, GERD, transoral incisionless fundoplication, TIF, EsophyX 2.0/Z, MUSE, GERD-X, anti-reflux mucosectomy, ARMS, anti-reflux mucosal ablation, ARMA

## Abstract

Transoral endoscopic therapies in gastro-esophageal reflux disease (GERD) are increasingly performed in patients who do not respond to medical therapy or are not suitable for or willing to undergo long-term PPI therapy or surgery. Currently available effective techniques include reconstruction of the gastro-esophageal valve by transoral incisionless fundoplication (TIF) and tightening of the gastro-esophageal junction through scarring, obtained by mucosal resection or ablation. TIF may be accomplished by an EsophyX 2.0/Z, MUSE, or GERD-X device. An iatrogenic stricture of the cardia may be obtained using a procedure called anti-reflux mucosectomy (ARMS), which includes several technical variants, or through mucosal ablation (ARMA). TIF using EsophyX 2.0 has strong evidence of efficacy in patients with small hiatal hernias, irrespective of hernia reducibility, who experience high-volume reflux episodes and troublesome regurgitation despite PPI therapy. MUSE can be performed only in the presence of a spontaneously reducing hiatal hernia and is probably more effective than EsophyX in maintaining the reduced hernia over time. However, MUSE is no longer available in Western countries. GERD-X shows promising results but needs further confirmation of its efficacy over the long term. ARMS and ARMA are not indicated in the presence of hiatal hernias but have shown promising results in the short term and are less expensive than TIF. Appropriate patient selection and the possibility of proposing a tailored approach to different types of patients and clinical/anatomical conditions result in favorable outcomes in most GERD patients, especially considering their quality of life and independence from PPIs. In the last several years, transoral endoscopic therapies have been proposed, along with concomitant laparoscopic repair for large hiatal hernias (cTIF), for GERD occurring after esophageal peroral endoscopic myotomy (E-POEM), in obese patients before or after bariatric surgery, and in patients with Barrett’s esophagus.

## 1. Introduction

Gastro-esophageal reflux disease (GERD) is a functional disorder with rising prevalence in Western countries. Proton pump inhibitors (PPIs) represent the standard therapy, in addition to lifestyle modifications. However, about 40% of patients suffer from symptom recurrence upon discontinuing PPIs and require long-term treatment, with potential side effects of prolonged acid suppression and a poor quality of life [1,2,3,4]. Notably, only about 50–60% of patients suffering from non-erosive reflux disease (NERD) benefit from PPIs, and many of them may have a hypersensitive esophagus or functional heartburn, which are less responsive to acid suppression [5]. Moreover, the healthcare and economic burden of long-term therapy with PPIs may be a concern in young patients [6].

For patients who suffer from refractory GERD or have contraindications to long-term use of PPIs, laparoscopic or transoral fundoplication may be required.

Surgical fundoplication creates a total 360° or a partial posterior 90° to 120° or anterior 180° wrap, depending on the patient’s characteristics and the surgeon’s expertise. However, outcomes are suboptimal and associated with risks of persistent side effects, affecting up to 30% of patients after total 360° (Nissen) fundoplication, which may discourage patients suffering from mild GERD from undergoing surgical treatment.

Transoral endoluminal therapy has been proposed to overcome the drawbacks of medical and surgical therapy, although unsatisfactory outcomes and safety issues were observed in the first years. However, since 2007, novel transoral techniques have been developed to reinforce the barrier function of the cardia, with proven efficacy in treating GERD in selected patients and an optimal safety profile.

Two main categories of transoral anti-reflux therapy are currently available:

1. Reconstruction of the gastro-esophageal valve by transoral incisionless fundoplication (TIF). The most commonly used techniques include EsophyX 2.0/Z (EndoGastric Solutions), MUSE (Medigus Ultrasonic Surgical Endostapler), and GERD-X (G-SURG GmbH).
2. Tightening of the gastro-esophageal junction through scarring. An iatrogenic stricture of the cardia may be obtained through mucosal resection, known as anti-reflux mucosectomy (ARMS), which includes a number of technical variants, or through mucosal ablation, known as anti-reflux mucosal ablation (ARMA).

In this narrative review, primary indicators of the clinical efficacy of endoscopic anti-reflux therapies were considered to be overall patient satisfaction, improvement in symptom scores (GERD-Health-Related Quality of Life, HRQL) and PPI discontinuation. Improvement in functional tests (total number of reflux episodes, % time pH < 4, and DeMeester score at pH-metry recording) was considered a secondary outcome when reported. Outcomes were evaluated at one to three years and >3 years post-intervention.

## 2. Methodology and Study Selection

To provide an up-to-date narrative review, we conducted a comprehensive literature search in the following databases: PubMed, Web of Science, and Scopus (from inception through November 2025). The search strategy used keywords relevant to the main focus areas: gastro-esophageal reflux disease, anti-reflux therapy, transoral incisionless fundoplication, anti-reflux mucosectomy, and anti-reflux mucosal ablation. The search included only peer-reviewed articles published in English, including original studies, meta-analyses and reviews. To overcome the potential overlapping of data published in different meta-analyses, the relative results are presented separately. Additional sources were identified through a manual review of the reference lists of key articles.

## 3. Transoral Incisionless Fundoplication (TIF)

Unlike laparoscopic fundoplication (LF), TIF restores the function of the lower esophageal sphincter (LES) without surgical incisions by creating a full-thickness 180° to 270° gastro-esophageal valve from inside the stomach, using serosa-to-serosa plications that include the muscle layers, depending on the adopted technique.

### 3.1. EsophyX 2.0/Z

EsophyX was approved by the FDA in 2007 and is the endoluminal anti-reflux procedure with the longest clinical experience. This technique reduces the angle of “His” and corrects small hiatal hernias by constructing a 250–300° (mean 270°) wrap, achieved by placing at least 20 fasteners across the esophago-gastric tissue, 1 and 3 cm below the Z-line. Once the tissue is engaged, a torque maneuver is performed, rotating the fundus of the stomach around the esophagus and increasing the success rate of the procedure by 30% [7,8,9].

TIF using EsophyX 2.0/Z reduces hiatal hernias ≤2.5 cm, and it is the only available transoral intervention that fulfills four of the five principles required for an effective anti-reflux surgery. It reduces hiatal hernias, elongates the intra-abdominal esophagus, approximates and tightens the gastric fundus around the esophagus, creating a fundoplication without strictures, and restores the gastro-esophageal high-pressure zone.

Adverse events have been reported in about 0.36% of cases in over 25,000 TIF 2.0 procedures performed since the year 2008, with no mortalities [10]. Severe complications have occurred in 2.5% of cases.

### 3.2. MUSE

MUSE is currently performed only in China and in some other Far East countries. This device mimics a 3 cm-long, 180° anterior fundoplication. It staples the gastric fundus to the esophagus below the diaphragm by placing three sets of five metal stitches each. The stapling procedure is carried out under ultrasonographic control and guided by software [11,12,13,14]. Differently from EsophyX, MUSE can reduce only reducible hiatal hernias ≤2.5 cm by applying a positive end-expiratory pressure of at least 5 mm Hg during the procedure.

The MUSE procedure is associated with a rate of severe adverse events, such as those of EsophyX 2.0, but more severe and life-threatening, requiring surgical repair in some cases. Severe complications included pneumothorax, esophageal leaks, and perforation [15]. Despite the reported good clinical outcomes, this technique has been adopted in a few centers in Western countries.

### 3.3. GERD-X

GERD-X is a modified model of the Plicator device. It creates an anterior 180° fundoplication. The device includes an applicator and a tissue retractor device, located inside the distal tip of the applicator. By using a flexible endoscope and a guidewire, the applicator is introduced into the stomach and retroflexed into the gastric fundus. Then, through a rotational movement, the Plicator engages the tissue of the cardia between the arms, deploying a pretied transmural pledged suture. The procedure can be repeated to obtain a tight closure [16,17]. Differently from EsophyX and MUSE, data on GERD-X’s capacity to reduce hiatal hernias ≤2.5 cm are still limited.

No major procedure-related complications have been reported so far; however, few patients have been treated using this technique.

## 4. Tightening of the Gastro-Esophageal Junction Through Scarring

This technique was discovered by Inoue and colleagues in 2003, when patients with Barrett’s esophagus reported GERD symptom relief after endoscopic mucosal resection (EMR), attributed to the scar shrinkage of the cardia.

A variety of endoscopic procedures aimed at tightening the gastro-esophageal junction have been developed over the years: anti-reflux mucosectomy (ARMS), banded anti-reflux mucosectomy (ARM-b), anti-reflux mucoplasty (ARM-P), peroral endoscopic cardiac constriction (PECC), anti-reflux mucosal valvulo-plasty (ARMV), anti-reflux ESD (ESD-G), and anti-reflux mucosal ablation (ARMA).

### 4.1. Anti-Reflux Mucosal Resection

ARMS was formally introduced in clinical practice in 2014 in a series of 10 GERD patients who reported significant improvement in pH-metric recordings and discontinued PPI use [18].

The procedure is carried out by performing an EMR along the lesser curvature, extending 1 cm into the esophagus and 2 cm into the stomach. The anti-reflux effect of ARMS is due to scar formation along the gastric side, resulting in a narrowed cardia opening. Initially, a circumferential EMR was performed, with effective reduction of refluxes but also with a high rate of strictures that required dilation. To avoid this complication, a mucosal resection involving half to two-thirds of the circumference was performed, achieving a good compromise between the efficacy in treating reflux symptoms and the risk of stricture [18,19,20].

Variants of ARMS include banded anti-reflux mucosectomy (ARM-b), anti-reflux mucoplasty (ARM-P), anti-reflux mucosal valvulo-plasty (ARMV), and peroral endoscopic cardiac constriction (PECC). ARM-b involves a piecemeal EMR of three-quarters of the circumference at the esophago-gastric junction, using the band ligation system and a snare [21]. In contrast, the ARM-P removes approximately one-third of the mucosal circumference along the lesser curvature of the gastric cardia through EMR [22]. ARMV creates an anti-reflux valve by utilizing the mobilized cardia mucosa. In this technique, the created ulcer is left open without closure, following a healing process similar to ARMS [23]. Together with ARM-b, the same authors proposed peroral endoscopic cardiac constriction (PECC), which consists of a mucosectomy at the esophago-gastric junction to create a long scar for a stronger anti-reflux effect [21].

Although several studies have demonstrated the efficacy of ARMS in reducing gastro-esophageal reflux and related symptoms, the mechanisms behind its effectiveness have not yet been investigated and remain unclear. Two hypotheses have been proposed in the only available study focusing on this point: the fibrosis occurring after ARMS tightens the esophago-gastric junction, or partial resection of the cardiac mucosa may result in the loss of stretch receptors in the gastric wall, with a possible decreased frequency of transient LES relaxations [24].

Around the same time that ARMS was introduced, endoscopic submucosal dissection for GERD (ESD-G) was reported [25]. The major difference between ARMS and ESD-G is the location of the created ulceration: in ARMS, it is placed at the gastric side, while in ESD-G, it is placed at the distal esophagus.

### 4.2. Anti-Reflux Mucosal Ablation (ARMA)

ARMA was introduced, again, by Inoue and colleagues to treat a patient in whom ARMS failed to close the cardia and GERD symptoms persisted. Ablation is initiated 0.5 to 1.0 cm proximal to the Z-line and extends distally to 2.0 cm below it, following the lesser curvature of the stomach.

In general, about 50% of the circumference is ablated, but the ablation can be extended to two-thirds in cases with low LES basal pressure or severe esophagitis. Inoue and colleagues reported a significant improvement in GERD-related symptoms and functional findings in 12 patients. Post-procedure stricture occurred only in one patient [26].

Few adverse events have been reported in both ARMS and its variants and ARMA in published studies. In total, after ARMS, a 17.2% pool rate of adverse events was reported, and most of them were self-limiting or manageable with conservative treatment. Esophageal stricture was the most frequent complication (11.4%), requiring endoscopic dilation in 10% of cases, and hemorrhage and perforation occurred in 2.2% of cases. A neuromuscular injury leading to aspiration pneumonia has also been reported in one case. Notably, the variants of ARMS did not appear to significantly influence the rate of adverse events. After ARMA, transient stenosis occurred in 13% of cases, all easily managed with endoscopic balloon dilation [27].

Neither ARMS nor ARMA can reduce hiatal hernias, but they have the advantage of being cheaper than other endoscopic device-assisted anti-reflux procedures.

## 5. Clinical Efficacy of Endoluminal Anti-Reflux Therapies

### 5.1. Transoral Fundoplication Techniques

The endoluminal platform with the greatest global clinical experience so far is TIF performed with EsophyX 2.0. Its efficacy at one to three years has been documented in two meta-analyses including 963 [28] and 1475 [29] patients, showing a median satisfaction rate of 72% (range 58–86%) [28], a significant improvement in GERD-related symptoms (*p* < 0.001) [29], and a mean PPI discontinuation rate of 59% [28] and 89% [29]. A significant reduction in the total number of reflux episodes (*p* < 0.00001) and DeMeester scores (*p* < 0.001) was reported, too. In three meta-analyses comparing TIF with magnetic sphincter augmentation (MSA) [30], MSA, STRETTA and LF [31], and MSA and LF [32], at one-year follow-up about 80% of patients reported satisfaction and PPI discontinuation [30] and GERD-HRQL scores improved significantly [32]. The total number of reflux episodes, % time pH < 4 and DeMeester score improved significantly, too. TIF outcomes were substantially comparable with those of other procedures, with TIF ranking highest for PPI discontinuation [31]. A recent prospective multicentre trial confirmed previous findings, reporting significant improvements in GERD-HRQL scores and PPI discontinuation in 89% and 80% of patients [33].

Data on the efficacy of MUSE are limited [11,34,35]. All studies reported a significant improvement in patient satisfaction and GERD-HRQL scores and a mean PPI discontinuation rate of 76% one to three years after the intervention, with rates similar to those of EsophyX 2.0, but with the MUSE series showing an 18.6% higher improvement in GERD-HRQL scores [15]. The total number of reflux episodes and the DeMeester score were significantly improved only in one study [14].

For GERD-X, data are limited, with follow-up reported only at 3 and 12 months [16,17]. Again, all studies reported significant improvements in the GERD-HRQL score, but outcomes assessed by objective parameters were limited. The largest study, carried out in 70 patients, reported at one year a significant improvement in GERD-HRQL score with 63% of patients off PPIs, while total reflux episodes did not improve [17]. To date, studies in more patients and with longer follow-up are required to confirm the clinical efficacy of GERD-X.

Long-term data have been reported mostly for the EsophyX 2.0 procedure. A meta-analysis reported mean satisfaction rates of 70% and 86% at 3 and 4–5 years, respectively, and 76% in a single report at 8–10 years [36]. Ten years after TIF, significant rates of 80% and 60% for improvement of quality of life and PPI discontinuation were still maintained. Five years after TIF performed by MUSE, satisfaction rates varied from 77% to 84% of patients, and PPI discontinuation rates from 54% to 85% of patients [11,13,14]. GERD recurrence was reported in 23% of cases [11].

These data are clinically relevant because they show that satisfactory post-TIF outcomes are maintained even in the long-term and seem to be similar to those of surgical fundoplication regarding symptom alleviation and PPI discontinuation, but without persistent side effects. However, it must be pointed out that comparative studies are difficult because of the different criteria of clinical success and endpoints reported. Substantially comparable long-term efficacy in controlling typical reflux symptoms and discontinuation of PPI use was confirmed in the meta-analysis comparing the different laparoscopic fundoplication techniques, MSA, STRETTA, TIF, and PPIs [30,31,32,37].

### 5.2. ARMS and ARMA

Outcomes of ARMS are generally limited to 3 years, with just one study reporting data at 5 years. Two Japanese studies reported discontinuation of PPI use in 40% and 50% of patients [38,39]. A prospective study carried out in China reported symptom improvement in 72.7% and 50% of patients at one and 2 years [40]. In a Korean series, 86% of patients showed improvement in GERD-HRQL scores, and 35% were able to discontinue PPI use, in a two-year follow-up [41]. A meta-analysis including 307 patients from 10 studies reported a pooled rate of 63% of patients who discontinued/reduced PPIs and a significant reduction in both GERD-HRQL score and mean acid exposure time after ARMS [42]. Five years after ARMS, satisfaction and discontinuation of PPI use were reported in 68.3% and 42% of patients, respectively. Interestingly, an 81% symptom-improvement rate was reported in patients with reflux hypersensitivity [39].

ARMA achieved a significant improvement in GERD-HRQL score and discontinuation of PPI in about 70% and 65% of patients, particularly in those with hypersensitive esophagus, but only three months after the procedure [43]

Overall, currently available data show that ARMS is better than ARMA in improving GERD symptoms, endoscopic findings, and PPI discontinuation. However, in a recent meta-analysis including 15 non-randomized studies (12 on ARMS with 331 patients, and 3 on ARMA with 130 patients), both ARMS and ARMA achieved at 12 and 36 months comparable improvement in GERD-HRQL scores (76% and 72%, respectively, at 36 months) and pooled rates of PPI discontinuation (61% and 68%, respectively, at 12 months) [44].

These data raise the question of whether ARMA could replace ARMS since both mucosal ablation and resection achieve similar GERD control, with fewer complications occurring with ablation. However, no comparative long-term studies have been carried out so far.

## 6. Which Patients Should Consider Endoluminal Therapy for GERD?

### 6.1. Indications for Endoluminal Therapy

In 2022, the clinical guidelines for GERD management published by the American Multi-Society Gastroenterological and Surgical Association agreed that TIF using EsophyX 2.0, ARMS, and MSA should be considered valid options for treating patients with GERD who do not respond to appropriate PPI therapy [45]. More recently, since 2025, the American Society of Gastrointestinal Endoscopy (ASGE) guidelines suggest considering TIF as an alternative to chronic medical therapy in patients with confirmed GERD, a small hiatal hernia (≤2.5 cm), and Hill grade I or II anatomy and who meet specific criteria [46].

Specific criteria for endoluminal therapy include proven GERD for at least 6 months, with at least partial response to PPIs; confirmed esophagitis (grade A or B according to the Los Angeles classification) or non-erosive esophagitis (NERD) with a hypersensitive esophagus documented during functional investigation; and absence of a hiatal hernia or with a hiatal hernia shorter than 2.5 cm. However, clinical response in patients with a hypersensitive esophagus appears to be more uncertain. Barrett’s esophagus ≤3 cm may also be an indication. Endoluminal therapy can also be proposed to patients who have contraindications to PPI use or require high-dose long-term therapy and refuse a surgical procedure.

Further parameters that should be assessed for appropriate anti-reflux therapy are as follows: (a) presence of a hiatal hernia, its vertical and axial sizes, and whether it is reducible; vertical extension up to 2.5 cm and an axial diameter up to 2.5 cm are acceptable for endoluminal therapy. Vertical extension can be better assessed by high-resolution manometry and axial diameter by an open biopsy forceps in retroflexed view or by CT scan. (b) The Hill’s grade of the gastro-esophageal junction; grades 1 or 2 of the valve can be successfully treated. (c) Whether the gastro-esophageal valve needs to be rebuilt; the length of the valve can be measured by endoscopy in retroflexed view. Generally, a length of 3 cm is considered to represent an effective valve, according to Jobe et al. [47]. (d) The need for tightening the right crura. (e) Whether there is an impairment of esophageal motility and its severity.

### 6.2. Choosing the Appropriate Technique for Each Patient

Among patients in whom endoscopic therapy is indicated, the presence of a hiatal hernia and its reducibility are the most important factors for a tailored approach.

In the presence of a hiatal hernia, irrespective of its spontaneous reducibility, TIF using EsophyX 2.0/Z represents the best therapeutic option. The device can reduce the hernia below the diaphragm and create a 3 cm-long new gastro-esophageal valve, which is further reinforced by the rotational technique. This neovalve is largely similar to that obtained by a 360° fundoplication but less strong. This aspect makes this technique an effective alternative to a 360° fundoplication in patients with documented ineffective esophageal motility. In addition to reducing the hernia, the EsophyX 2.0/Z procedure elongates the intra-abdominal esophagus, recreates the dynamics of the angle of His, and recovers the gastro-esophageal high-pressure segment.

In the presence of a hiatal hernia that reduces spontaneously, the MUSE technique can be used as an alternative to EsophyX 2.0/Z. With this technique, a fundoplication can be performed only once the hernia has been reduced below the diaphragm by applying PEEP in an intubated patient. This neovalve is less closed (180°) but stronger and more efficient in treating hiatal hernias compared to the EsophyX 2.0/Z technique (88.9% vs. 51.9% at one year) [15]. With the MUSE device, the length of the neovalve rather than its pressure creates a barrier to reflux. However, despite the greater efficacy of the MUSE system in reducing hiatal hernias, the rate of esophagitis was found to be unexpectedly higher and unrelated to symptomatic improvement compared with that of EsophyX 2.0 [15]. This may be due to impaired distal esophageal clearance depending on the longer neovalve obtained with the MUSE system. The complication rate of the two techniques is similar, but with EsophyX 2.0, complications were less severe and possibly preventable by an expert operator, while with MUSE, they were more severe, life-threatening in some cases, and not preventable by the operator.

In GERD patients without a hiatal hernia, ARMS and its variants can be successfully used as an alternative to TIF, because they are unable to effectively treat a sliding hiatal hernia. ARMS has been shown to be particularly effective in patients with reflux hypersensitivity, with an 81% response rate. Compared with TIF, ARMS has lower costs because it does not require dedicated expensive devices, but is very operator-dependent, leading to wide variations in outcomes across various centers. Overall, the outcomes of ARMS are worse than those reported for TIF: at one- and five-year follow-ups, PPI discontinuation was reported in 65.3% and 85.7% and in 57.5% and 75.8% of patients undergoing ARMS and EsophyX 2.0, respectively [48]. The overall 17% reported complication rate for ARMS was higher than that for TIF, with 10% of patients suffering from esophageal strictures requiring dilation.

In cases of unsuccessful ARMS, ARMA could be the preferred second treatment because the submucosal fibrosis induced by the previous mucosal resection makes ARMS technically challenging.

Selective indications for the different endoscopic anti-reflux procedures, on the basis of the literature data, are synthesized in Table 1.

## 7. Future Perspectives for Endoscopic Anti-Reflux Interventions

As regards future perspectives, it should be noted that the most important critical issue of transoral anti-reflux interventions concerns their long-term efficacy, due to the lack of prospective comparative trials versus LF techniques, which are still considered the gold standard therapy. However, conducting such trials is practically impossible because patients who consider an endoscopic intervention typically rule out the option of surgery. In fact, transoral and laparoscopic interventions presumably apply to different types of patients, with varying degrees of disease severity, so current comparisons do not provide reliable information.

Another unsettled issue is whether TIF is capable of treating atypical GERD symptoms, which are reported to be increasing and mostly occur in the absence of a hiatal hernia and esophagitis. Data from the only available meta-analysis, including 564 patients, reported satisfaction and PPI discontinuation rates of 73% and 74%, respectively, at one year [49].

Besides this consideration, TIF using EsophyX 2.0/Z has been proposed in combination with laparoscopic hernia repair (cTIF), after peroral esophageal endoscopic myotomy (E-POEM), and before or after gastric restrictive interventions or Roux-en-Y (RYGB) surgery for obesity.

cTIF can be performed during laparoscopic repair of large hernias to prevent the possible postoperative side effects induced by the creation of a fundoplication around the gastric fundus. In 2017, the Food and Drug Administration (FDA) approved cTIF in patients with a hiatal hernia larger than 2 cm. Available reports show that after cTIF, 74% to 90% of subjects discontinued PPIs without experiencing adverse events related to laparoscopic surgery [50].

TIF can be successfully performed after E-POEM. After E-POEM, as well as after Heller myotomy, about 50% of patients develop GERD, but this risk is reduced to 10% if a laparoscopic partial fundoplication is performed concomitantly with the myotomy. Performing TIF only in cases of post-POEM GERD rather than routinely performing fundoplication in all patients undergoing Heller myotomy—when GERD only occurs in about 50% of cases—would be a more cost-effective strategy. TIF has also been used in type 1 achalasia with dilated and tortuous esophagus to fix the esophagus below the diaphragm and to straighten it before E-POEM, facilitating the tunneling technique.

Obese patients have a higher incidence of GERD compared with non-obese subjects, and both laparoscopic sleeve gastrectomy and endoscopic gastroplasty increase the occurrence of GERD. Therefore, in obese patients suffering from GERD and candidates for gastric restrictive interventions, TIF can be proposed to avoid the risk of a subsequent RYGB procedure.

ARMA is the preferred endoscopic intervention for treating GERD occurring after gastric restrictive surgery or RYGB for obesity, because these interventions reduce the space in the gastric fundus, making it difficult or impossible to use retroflexed instruments, including ARMS [51]. ARMA represents a cost-effective approach, allowing treatment only for patients who develop GERD after bariatric surgery and avoiding more invasive interventions.

The efficacy of endoluminal anti-reflux procedures has not yet been investigated in patients with Barrett’s esophagus, and only a few cases with short-segment disease have been included in the protocol studies, without information about long-term outcomes. However, it seems reasonable to strengthen and maintain the anti-reflux barrier over time in these patients, together with appropriate PPI therapy, since other factors besides hydrochloric acid are involved in the growth of columnar epithelium. In particular, endoscopic anti-reflux procedures could be offered to patients who have achieved successful endoscopic eradication of Barrett’s epithelium and need lifelong PPI therapy.

In conclusion, both TIF and ARMS/ARMA improve GERD symptoms and reduce medication use. TIF can effectively treat patients with small hiatal hernias and provides greater long-term PPI reduction and better quality of life compared with ARMS. TIF using EsophyX 2.0/Z can also be performed along with a concomitant laparoscopic fundoplication. ARMS appears to be suitable for patients without a hiatal hernia, particularly for those seeking efficient symptom control, including patients suffering from a hypersensitive esophagus. ARMA could be performed in cases of ARMS failure and after gastric restrictive surgery for obesity.

## Figures and Tables

**Table 1:** Selective indications for the transoral anti-reflux procedures, based on the current literature data.

Procedure	GERD with Typical Symptoms	GERD with Atypical Symptoms	Hiatal Hernia <2.5 cm Irrespective of Reducibility	Hiatal Hernia <2.5 cm Reducible	No Hiatal Hernia	Hyper- Sensitive Esophagus	Post-Bariatric Procedures	Post- E-POEM
EsophyX 2.0/Z	++	+	++	++	++	-	NA	+
MUSE	++	-	NA	++	++	-	NA	-
GERD-X	+	-	NA	+	+	-	NA	-
ARMS + variations	++	-	NA	NA	++	++	NA	-
ARMA	+	-	NA	NA	+	+	+	-

GERD: gastro-esophageal reflux disease; E-POEM: esophageal peroral endoscopic myotomy; MUSE: Medigus Ultrasound Surgical Endostapler; ARMS: anti-reflux mucosal resection; ARMA: anti-reflux mucosal ablation. ++: effective documentation from several meta-analyses; +: effective documentation from few studies/single meta-analysis; -: insufficient or lack of documentation; NA: not applicable (lack of indications)

## Data Availability

No new data were created or analyzed in this study.

## References

[1] Sharma N., Agrawal A., Freeman J., Vela M.F., Castell D. (2008). An analysis of persistent symptoms in acid-suppressed patients undergoing impedance-pH monitoring. Clin. Gastroenterol. Hepatol..

[2] Hershcovici T., Fass R. (2010). An algorithm for diagnosis and treatment of refractory ERD. Best Pract. Res. Clin. Gastroenterol..

[3] Cicala M., Emerenziani S., Guarino M.P.L., Ribolsi M. (2013). Proton pump inhibitor resistance, the real challenge in gastro-esophageal reflux disease. World J. Gastroenterol..

[4] Rettura F., Bronzini F., Campigotto M., Lambiase C., Pancetti A., Berti G., Marchi S., de Bortoli N., Zerbib F., Savarino E. (2021). Refractory gastroesophageal reflux disease: A management update. Front. Med..

[5] Young A., Kumar M.A., Thota P.N. (2020). GERD: A practical approach. Clev. Clin. J. Med..

[6] Faria R., Bojke L., Epstein D., Corbacho B., Sculpher M. (2013). REFLUX trial group. Costeffectiveness of laparoscopic fundoplication versus continued medical management for the treatment of gastro-oesophageal reflux disease based on long-term follow-up of the REFLUX trial. Br. J. Surg..

[7] Cadière G.B., Rajan A., Rquibate M., Germay O., Dapri G., Himpens J., Gawlicka A.K. (2006). Endoluminal Fundoplication (ELF)—Evolution of EsophyX™, a new surgical device for transoral surgery. Minim. Invasive Ther. Allied Technol..

[8] Bell R.C., Cadiere G.B. (2011). Transoral rotational esophagogastric fundoplication: Technical, anatomical, and safety considerations. Surg. Endosc..

[9] Testoni P.A., Mazzoleni G., Testoni S.G.G. (2016). Transoral incisionless fundoplication for gastroesophageal reflux disease: Techniques and outcomes. World J. Gastrointest. Pharmacol. Ther..

[10] Ramai D., Shapiro A., Barakat M., Facciorusso A., Dull A., Chandan S., Adler D.G. (2022). Adverse events associated with transoral incisionless fundoplication (TIF) for chronic gastroesophageal reflux disease: A MAUDE database analysis. Surg. Endosc..

[11] Roy-Shapira A., Bapaye A., Date S., Pujari R., Dorwat S. (2015). Trans-oral anterior fundoplication: 5year follow-up of pilot study. Surg. Endosc..

[12] Zacherl J., Roy-Shapira A., Bonavina L., Bapaye A., Kiesslich R., Schoppmann S.F., Kessler W.R., Selzer D.J., Broderick R.C., Lehman G.A. (2015). Endoscopic anterior fundoplication with the Medigus Ultrasonic Surgical Endostapler (MUSE) for gastroesophageal reflux disease: 6-month results from a multi-center prospective trial. Surg. Endosc..

[13] Testoni P.A., Testoni S., Mazzoleni G., Pantaleo G., Cilona M.B., Distefano G., Fanti L., Antonelli M., Passaretti S. (2020). Transoral incisionless fundoplication with an ultrasonic surgical endostapler for the treatment of gastro-esophageal reflux disease: 12-month outcomes. Endoscopy.

[14] Testoni S.G.G., Cilona M.B., Mazzoleni G., Fanti L., Ribichini E., Cavestro G.M., Esposito D., Viale E., Notaristefano C., Zuppardo R.A. (2022). Transoral incisionless fundoplication with Medigus ultrasonic surgical endostapler (MUSE) for the treatment of gastro-esophageal reflux disease: Outcomes up to 3 years. Surg. Endosc..

[15] Testoni S.G.G., Pantaleo G., Contu F., Azzolini F., Fanti L., Testoni P.A. (2024). Comparison of EsophyX2.0 and MUSE systems for transoral incisionless fundoplication: Technical aspects and outcomes up to 3 years. Dig. Endosc..

[16] Weitzendorfer M., Spaun G.O., Antoniou S.A., Witzel K., Emmanuel K., Koch O.O. (2018). Clinical feasibility of a new full-thickness endoscopic plication device (GERDxTM) for patients with GERD: Results of a prospective trial. Surg. Endosc..

[17] Kalapala R., Karyampudi A., Nabi Z., Darisetty S., Jagtap N., Ramchandani M., Rajesh G., Rao V., Sharma P., Reddy D.N. (2022). Endoscopic full-thickness plication for the treatment of PPIdependent GERD: Results from a randomised, sham controlled trial. Gut.

[18] Inoue H., Ito H., Ikeda H., Sato C., Sato H., Phalanusitthepha C., Hayee B., Eleftheriadis N., Kudo S.E. (2014). Anti-reflux mucosectomy for gastroesophageal reflux disease in the absence of hiatus hernia: A pilot study. Ann. Gastroenterol..

[19] Wong H.J., Su B., Attaar M., Kucht K., Stearns S., Linn J.G., Haggert S.P., Denham W., Ujiki M.B. (2021). Anti-reflux mucosectomy (ARMS) results in improved recovery and similar reflux quality of life outcomes compared to laparoscopic Nissen fundoplication. Surg. Endosc..

[20] Zhu X., Shen J. (2024). Anti-reflux mucosectomy (ARMS) for refractory gastroesophageal reflux disease. Eur. J. Med. Res..

[21] Monino L., Gonzalez J.M., Vitton V., Barthet M. (2019). Anti-reflux mucosectomy with band ligation in the treatment of refractory gastroesophageal reflux disease. Endoscopy.

[22] Inoue H., Yamamoto K., Shimamura Y., Azuma D., Ushikubo K., Okada H., Kimoto Y., Nishikawa Y., Tanaka I., Tanabe M. (2024). Pilot study on anti-reflux mucoplasty: Advancing endoscopic anti-reflux therapy for gastroesophageal reflux disease. Dig. Endosc..

[23] Lu X., Ma W., Zeng Y., Lu J. (2024). Antireflux mucosal valvuloplasty versus proton pump inhibitors for the treatment of patients with gastro-oesophageal reflux disease in a tertiary healthcare centre in China: Study protocol for a randomised controlled trial. BMJ Open.

[24] Thijs K., Oude Nijhuis R.A.B., Pouw R.E., Bredenoord A.G. (2024). Anti-reflux mucosectomy for gastroesophageal reflux disease: Efficacy and the mechanism of action. Endoscopy.

[25] Ota K., Takeuchi T., Harada S., Edogawa S., Kojima Y., Inoue T., Higuchi K. (2014). A novel endoscopic submucosal dissection technique for proton pump inhibitor-refractory gastroesophageal reflux disease. Scand. J. Gastroenterol..

[26] Inoue H., Tanabe M., de Santiago E.R., Angeli Abad M.R., Shimamura Y., Fujiyoshi Y., Ueno A., Sumi K., Tomida I., Iwaya Y. (2020). Antireflux mucosal ablation (ARMA) as a new treatment for gastroesophageal reflux refractory to proton pump inhibitors: A pilot study. Endosc. Int. Open.

[27] Yeh J.H., Lee C.T., Hsu M.H., Lin C.W., Hsiao P.J., Chen C.L., Wang W.L. (2022). Antireflux mucosal intervention (ARMI) procedures for refractory gastroesophageal reflux disease: A systematic review and meta-analysis. Therap. Adv. Gastroenterol..

[28] Huang X., Chen S., Zhao H., Zen X., Lian X., Tseng Y., Chen J. (2017). Efficacy of transoral incisionless fundoplication (TIF) for the treatment of GERD: A systematic review with meta-analysis. Surg. Endosc..

[29] McCarty T.R., Itidiare M., Njei B., Rustagi T. (2018). Efficacy of transoral incisionless fundoplication for refractory gastroesophageal reflux disease: A systematic review and meta-analysis. Endoscopy.

[30] Chandan S., Mohan B.P., Khan S.R., Jha L.K., Dhaliwal A.J., Bilal M., Aziz M., Canakis A., Arora S., Malik S. (2021). Clinical efficacy and safety of magnetic sphincter augmentation (MSA) and transoral incisionless fundoplication (TIF2) in refractory gastroesophageal reflux disease (GERD): A systematic review and meta-analysis. Endosc. Int. Open.

[31] Pasam R.T., Osman K.T., Mohan B.P., Adler D.G. (2023). Endoscopic versus surgical therapies for GERD: A systematic review and network meta-analysis. Gastrointest. Endosc..

[32] Tadé Y., Newman D., Walters R.W., Nandipati K.C. (2025). Fundoplication significantly improves objective and subjective reflux outcomes-a meta-analysis. Surg. Endosc..

[33] Canto M.I., Diehl D.L., Parker B., Abu-Dayyeh B.K., Kolb J.M., Murray M., Sharaiha R.Z., Brewer Gutierrez O.I., Sohagia A., Khara H.S. (2025). Outcomes of transoral incisionless fundoplication (TIF 2.0): A prospective multicenter cohort study in academic and community gastroenterology and surgery practices (with video). Gastrointest. Endosc..

[34] Kim H.J., Kwon C.I., Kessler W.R., Selzer D.J., McNulty G., Bapaye A., Bonavina L., Lehman G.A. (2016). Long-term follow-up results of endoscopic treatment of gastroesophageal reflux disease with the MUSE endoscopic stapling device. Surg. Endosc..

[35] Shen S., Yu G., Guo X., Zong G., Wang C., Bao J., Chen J., Cheng Z., Xiao W., Shen J. (2023). The long-term efficacy of transoral incisionless fundoplication with Medigus Ultrasonic Surgical Endostapler (MUSE) for gastroesophageal reflux disease. Esophagus.

[36] Testoni S., Hassan C., Mazzoleni G., Antonelli G., Fanti L., Passaretti S., Correale L., Cavestro G.M., Testoni P.A. (2021). Long-term outcomes of transoral incisionless fundoplication for gastroesophageal reflux disease: Systematic-review and meta-analysis. Endosc. Int. Open.

[37] Rausa E., Ferrari D., Kelly M.E., Aiolfi A., Vitellaro M., Rottoli M., Bonitta G., Bona D. (2023). Efficacy of laparoscopic Toupet fundoplication compared to endoscopic and surgical procedures for GERD treatment: A randomized trials network meta-analysis. Langenbecks Arch. Surg..

[38] Sumi K., Inoue H., Kobayashi Y., Iwaya Y., Abad M.R.A., Fujiyoshi Y., Shimamura Y., Ikeda H., Onimaru M. (2021). Endoscopic treatment of proton pump inhibitor refractory gastroesophageal reflux disease with anti-reflux mucosectomy: Experience of 109 cases. Dig. Endosc..

[39] Sumi K., Inoue H., Ando R., Iwaya Y., Angeli Abad M.R., Fujiyoshi Y., Shimamura Y., Ikeda H., Onimaru M. (2024). Long-term efficacy of antireflux mucosectomy in patients with refractory gastroesophageal reflux disease. Dig. Endosc..

[40] Hu J.N., Chen S.F., Jia X.Y., Luo Y., Xing X.B., Tan N.D., Zhang M.Y., Zhuang Q.J., Wang J.H., Xiao Y.L. (2023). Two-year outcomes of anti-reflux mucosectomy in treating gastroesophageal reflux disease: A Chinese prospective cohort study. J. Dig. Dis..

[41] Lee Y.S., Kim J.H., Yon D.K., Yoo I.K. (2024). Short- and long-term effect of anti-reflux mucosectomy with cap-assisted endoscopic mucosal resection for refractory gastroesophageal disease. Surg. Endosc..

[42] Garg R., Mohammed A., Singh A., Schleicher M., Thota P.N., Rustagi T., Sanaka M.R. (2022). Anti-reflux mucosectomy for refractory gastroesophageal reflux disease: A systematic review and metaanalysis. Endosc. Int. Open.

[43] Chen C.C., Chou C.K., Yuan M.C., Tsai K.F., Wu J.F., Liao W.C., Chiu H.M., Wang H.P., Wu M.S., Tseng P.H. (2025). Effect of anti-reflux mucosal ablation on esophageal motility in patients with gastroesophageal reflux disease: A study based on high-resolution impedance manometry. J. Neurogastroenterol. Motil..

[44] Rodríguez S.E., Sanchez-Vegazo C.T., Peñas B., Shimamura Y., Tanabe M., Álvarez-Díaz N., Parejo S., Kazuya S., Marcos-Carrasco N., Vazquez-Sequeiros E. (2021). Antireflux mucosectomy (ARMS) and antireflux mucosal ablation (ARMA) for gastroesophageal reflux disease: A systematic review and meta-analysis. Endosc. Int. Open.

[45] Slater B.J., Collings A., Dirks R., Gould J.C., Qureshi A.P., Juza R., Rodríguez-Luna M.R., Wunker C., Kohn G.P., Kothari S. (2023). Multisociety consensus conference and guideline on the treatment of gastroesophageal reflux disease (GERD). Surg. Endosc..

[46] Desai M., Ruan W., Thosani N.C., Amaris M., Scott J.S., Saeed A., Abu Dayyeh B., Canto M.I., Abidi W., ASGE Standards of Practice Committee (2025). American Society for Gastrointestinal Endoscopy guideline on the diagnosis and management of GERD: Summary and recommendations. Gastrointest. Endosc..

[47] Jobe B.A., O’Rourke R.W., McMahon B.P., Gravesen F., Lorenzo C., Hunter J.G., Bronner M., Kraemer S.J. (2008). Transoral endoscopic fundoplication in the treatment of gastroesophageal reflux disease: The anatomic and physiologic basis for reconstruction of the esophagogastric junction using a novel device. Ann. Surg..

[48] Mohan A., Sohail F., Saeed N.A., Jameel M., Assal M.W., Hasan Z.W., Zafar A., Aminpoor H., Kumar V. (2025). A comparative analysis of ARMS (anti-reflux mucosectomy) and TIF (transoral incisionless fundoplication) in the treatment of gastroesophageal reflux disease (GERD). Ann. Med. Surg..

[49] Haseeb M., Glissen Brown J.R., Hayat U., Bay C., Paul A., Bain P.A., Jirapinyo P., Thompson C.C. (2023). Impact of second-generation transoral incisionless fundoplication on atypical GERD symptoms: A systematic review and meta-analysis. Gastrointest. Endosc..

[50] Jaber F., Ayyad M., Ayoub F., Patel K.K., Makris K.I., Hernaez R., Skef W. (2024). Concomitant hiatal hernia repair with transoral incisionless fundoplication for the treatment of refractory gastroesophageal reflux disease: A systematic review. Surg. Endosc..

[51] Ayoub F., Patel K.K. (2024). Anti-reflux mucosal ablation for refractory gastroesophageal reflux disease after Roux-en-Y gastric bypass. Endoscopy.

